# Longitudinal Associations Between Hand Grip Strength and Non-Alcoholic Fatty Liver Disease in Adults: A Prospective Cohort Study

**DOI:** 10.3389/fmed.2021.752999

**Published:** 2021-10-29

**Authors:** Yang Xia, Limin Cao, Yashu Liu, Xuena Wang, Shunming Zhang, Ge Meng, Qing Zhang, Li Liu, Hongmei Wu, Yeqing Gu, Yawen Wang, Tingjing Zhang, Xing Wang, Shaomei Sun, Ming Zhou, Qiyu Jia, Kun Song, Kaijun Niu, Yuhong Zhao

**Affiliations:** ^1^Department of Clinical Epidemiology, Shengjing Hospital of China Medical University, Shenyang, China; ^2^The Third Central Hospital of Tianjin, Tianjin, China; ^3^Nutritional Epidemiology Institute and School of Public Health, Tianjin Medical University, Tianjin, China; ^4^Department of Toxicology and Sanitary Chemistry, School of Public Health, Tianjin Medical University, Tianjin, China; ^5^Health Management Centre, Tianjin Medical University General Hospital, Tianjin, China; ^6^Tianjin Key Laboratory of Environment, Nutrition and Public Health, Tianjin, China; ^7^Center for International Collaborative Research on Environment, Nutrition and Public Health, Tianjin, China

**Keywords:** hand grip strength, non-alcoholic fatty liver disease, cohort, China, general adults

## Abstract

**Purpose:** This study aimed to determine the longitudinal association between hand grip strength (HGS) and the development of non-alcoholic fatty liver disease (NAFLD) in adults.

**Design:** A cohort study.

**Methods:** This study was conducted in a general Chinese population (*n* = 14,154) from 2013–2018. NAFLD was diagnosed by liver ultrasonography during evaluating alcohol consumption. The associations between the HGS and NAFLD were assessed using a multivariable Cox proportional hazards regression model.

**Results:** During the study period with a mean follow-up duration of 3.20 years, 2,452 participants developed NAFLD. The risk of NAFLD decreased progressively with increasing HGS in both men and women (*P* for trend <0.0001). The multivariate-adjusted hazard ratios (95% *CI*) for NAFLD incidence across the quartiles of HGS were 1 (reference), 0.90 (0.79, 1.02), 0.69 (0.60, 0.79), and 0.44 (0.37, 0.52) for men and 1 (reference), 0.82 (0.69, 0.96), 0.54 (0.45, 0.66), and 0.41 (0.33, 0.52) for women, respectively. The interaction terms for body mass index (BMI)-HGS and waist-HGS were significant in men and women (all *P* < 0.0001). The participants with normal BMIs and waist circumferences had the lowest hazard ratios on the subgroup analyses. The sensitivity analysis that defined NAFLD using the hepatic steatosis and fatty liver indices revealed results that were similar to the main analyses.

**Conclusion:** The present study indicates that the HGS is inversely associated with the incidence of NAFLD.

## Introduction

The non-alcoholic fatty liver disease (NAFLD) represents a spectrum of liver diseases not attributable to alcohol consumption, such as simple fatty infiltration, inflammation, and cirrhosis. Globally, the NAFLD is one of the most important causes of liver disease (1) and the previous studies have demonstrated that it is associated with metabolic syndrome (2), diabetes (3), and hypertension (4). As reported in a meta-analysis conducted in 2016, 25% of the global adult population were afflicted with NAFLD (5). Moreover, in China, the prevalence of NAFLD among adults in the general population is >20% and has paralleled the increase in obesity (6). In addition, the obesity prevalence rose from 3.1% (2.5–3.7) in 2004 to 8.1% (7.6–8.7) in 2018 (7). In parallel with increasing prevalence, the economic burden of NAFLD is enormous, especially at the time of diagnosis (8). Therefore, it is important to identify the modifiable risk factors and develop preventive strategies.

Insulin resistance is shown to be an important factor in NAFLD progression (9). A muscle is a target organ for insulin (10), and the previous studies suggested that the skeletal muscles secrete a variety of metabolically bioactive factors, such as myostatin, interleukin-6, and irisin (11, 12) that are subsequently involved in the regulation of insulin resistance and lipid metabolism. Thus, it is plausible that the muscles play an important role in the development of NAFLD. Indeed, several cross-sectional studies have shown that muscle strength is associated with NAFLD (13–18). For example, a cross-sectional study involving 5,132 adults in China showed that the low muscle strength was positively and independently associated with NAFLD [odds ratio (*OR*), 1.47; 95% *CI*, 1.21, 1.80] (13). Another nationwide population-based cross-sectional study demonstrated that the high hand grip strength (HGS) was negatively associated with the hepatic steatosis index (HSI) in 4,764 participants of Koreas (14). Because of the cross-sectional design of these studies, however, a causal relationship could not be identified.

To our knowledge, there has been no cohort study conducted to investigate the associations between muscle strength and the incidence of NAFLD. Thus, we conducted this prospective study to better understand the association between the HGS and NAFLD using data from a large population-based cohort study in adults in China.

## Methods

### Participants

**Figure 1:**
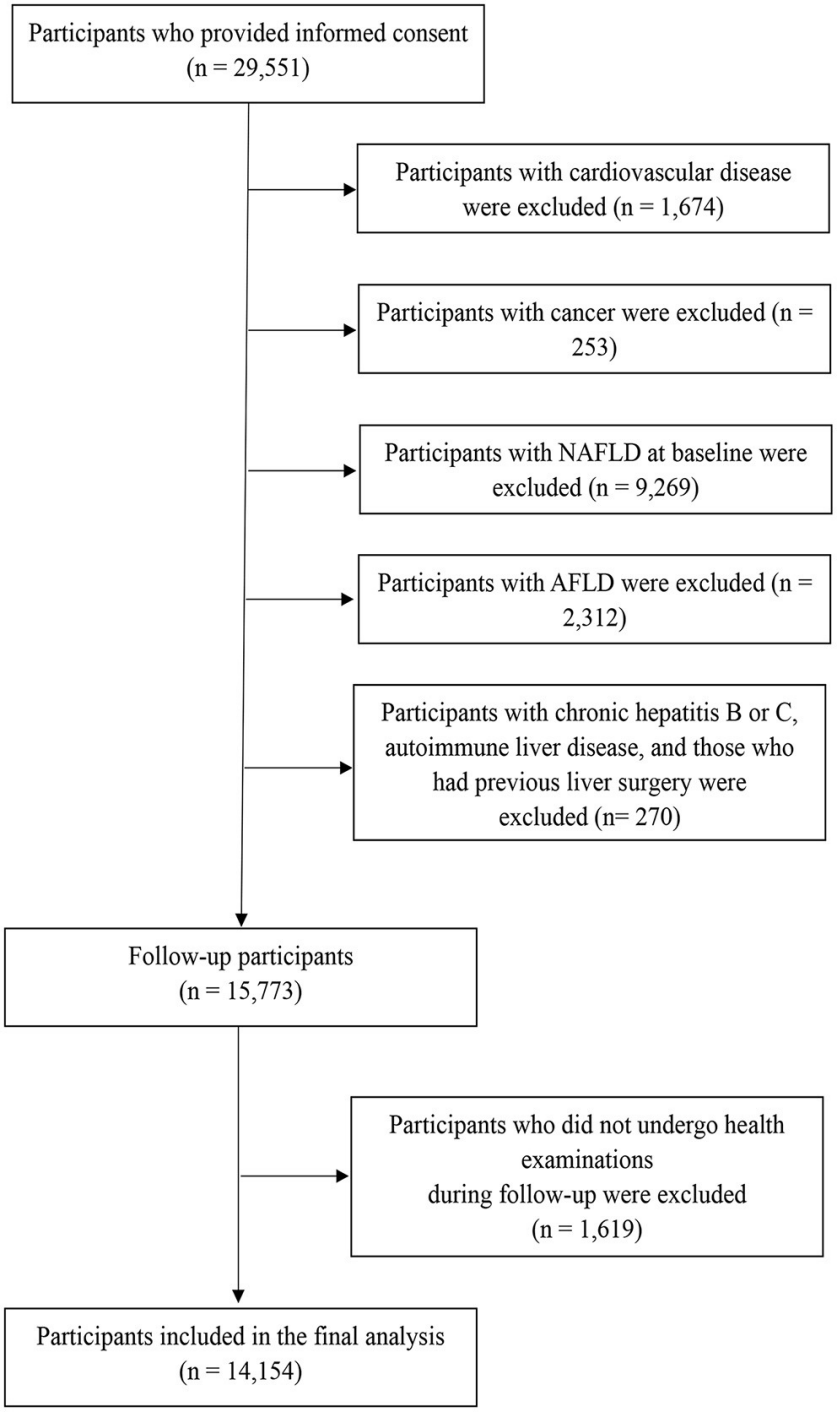
Flowchart of study participant selection for the study. AFLD, alcoholic fatty liver disease; NAFLD, non-alcoholic fatty liver disease.

This prospective study was based on a large prospective dynamic cohort study conducted in Tianjin, China (19). Between 2013 and 2018, a total of 29,551 participants had at least two health examinations with adequate data related to the NAFLD diagnosis, lifestyle (*via* questionnaire), and physical performance tests. After exclusions (Figure 1), the cohort consisted of 15,773 participants at baseline. As 1,619 participants did not complete the follow-up health examinations, the final study population comprised 14,154 participants (follow-up rate of 89.74%). The mean duration of follow-up was 3.20 years (range, 0.50–5 years). The protocol of this study was approved by the Institutional Review Board of the Tianjin Medical University. The subjects provided the written informed consent to participate in the study. The study protocol conformed to the ethical guidelines of the 1975 Declaration of Helsinki.

### Assessment of NAFLD

Liver ultrasonography was performed by the trained sonographers using a Toshiba SSA-660A instrument (Toshiba, Tokyo, Japan) with a 2–5 MHz curved array probe. According to the revised 2018 NAFLD definition and treatment guidelines promulgated by the Chinese National Workshop on Fatty Liver and Alcoholic Liver Disease (20), the participants were diagnosed with NAFLD based on the detection of brightness in the liver and a diffusely echogenic change in the liver parenchyma on abdominal ultrasonography despite no history of heavy alcohol consumption (defined as >210 g of alcohol intake per week in men and >140 g per week in women).

In the sensitivity analysis, the participants were diagnosed with NAFLD using the HSI and fatty liver index (FLI), which were developed to identify the presence of suspected NAFLD. The HSI was calculated using the following algorithm: HSI = 8 × the alanine aminotransferase-to-aspartate transaminase ratio + the body mass index [BMI] (+ 2, if type 2 diabetes was present and +2 if female). An HSI value >36.0 predicted the NAFLD with a specificity of 92.4% (95% *CI*, 91.3–93.4) (21). The FLI was calculated using the following algorithm: FLI = (e^0.953*loge (*triglycerides*) + 0.139*BMI + 0.718*loge (*ggt*) + 0.053*waistcircumference−15.745^)/ (1 + e^0.953*loge (*triglycerides*) + 0.139*BMI + 0.718*loge (*ggt*) + 0.053*waist circumference −15.745^)*100 (22). According to a previous study that explored the validation of the FLI for NAFLD in the Chinese, an FLI value ≥ of 30 was used as the cut-off point for NAFLD with a sensitivity of 79.89% and a specificity of 71.51% (23).

### Measurement of Muscle Strength

The muscle strength was assessed using the HGS, which is a feasible and convenient indicator of the overall muscle strength with good test-retest reliability and responsiveness (24). The participants were tested by the trained technicians using a handheld dynamometer (EH101; Camry, Guangdong, China). The participants were asked to stand upright with the dynamometer beside but not against their bodies and to perform two maximum force trials for each hand; the greatest force was used as the final score. Furthermore, HGS was normalized to the bodyweight to account for the proportion of HGS relative to the body weight [HGS (kg)/body weight (kg)] (25).

### Assessments and Definitions of Other Variables

The sociodemographic variables, such as sex, age, education, employment, and household income, were assessed *via* questionnaire, as were cigarette smoking and alcohol consumption. The BMI was calculated as the weight in kilograms divided by the square of the height in meters (kg/m^2^). The physical activity in the most recent week was assessed using the short form of the International Physical Activity Questionnaire (26). High depressive symptoms were assessed using the Chinese version of the Zung Self-Rating Depression Scale (SDS), a useful and well-validated questionnaire commonly used by the Chinese psychiatrists; (27) the participants were defined as high depressive symptoms when the SDS score was ≥45 (28). Hypertension was defined as an average systolic blood pressure ≥140 mm Hg or average diastolic blood pressure ≥90 mmHg or as the use of antihypertension medication (29). Hyperlipidemia was defined as a total cholesterol level ≥5.20 mmol/L, a triglycerides level ≥1.70 mmol/L, or as a self-reported clinical diagnosis of hyperlipidemia according to the 2016 Chinese guidelines for the management of dyslipidemia in adults (30). Dietary intake was assessed using a modified version of the Food Frequency Questionnaire (FFQ) that included 100 food items with specified serving sizes; detailed information about this FFQ has been described elsewhere (31, 32). The Chinese Food Composition tables were used as the nutrient database to calculate the total energy intake per day (33). The factor analysis was applied to generate the major dietary patterns and food loading for all the 100 food items and beverages in grams. The factors were named descriptively according to the food items showing high loading (absolute value > 0.3) with respect to each dietary pattern as follows: sweet foods pattern, vegetable pattern, and animal foods pattern. The dietary patterns scores were used for further analyses as confounding factors.

### Statistical Analysis

The characteristics of participants at the baseline are described according to sex and NAFLD status. The continuous variables are presented as least-square means and 95% *CI*, in which the categorical variables are presented as percentages. The quartiles were categorized across HGS based on the distribution of the scores and used for further analyses. The Cox proportional hazards regression model was used to estimate the hazard ratios (HRs) and 95% *CI*s for NAFLD incidence in relation to the HGS. The linear trend across increasing the quartiles of HGS was tested using the median value of each quartile as a continuous variable based on the Cox proportional hazards regression analysis. The crude model was used to calculate the crude HR without any adjustment. Model 1 adjusted for age and BMI. Model 2 additionally adjusted for cigarette smoking status, alcohol consumption status, educational level, employment status, household income, physical activity, energy intake, type 2 diabetes, hypertension, hyperlipidemia, depressive symptoms, and intake of sweet foods pattern, vegetable pattern, and animal foods pattern. The receiver operating characteristics (ROCs) curves were performed to quantify the area under the curve (AUC) and an optimal cut-off value of HGS associated with the incidence of NAFLD.

To study the BMI–HGS and waist–HGS interactions, the analyses according to different subgroups of BMI and waist circumferences were performed. The subgroups of BMI and waist circumferences were defined according to the Working Group on Obesity in China (normal BMI, <24 kg/m^2^; high BMI, 24–28 kg/m^2^; and obesity, ≥28 kg/m^2^ and normal waist, <80 cm for women and <85 cm for men; and high waist, ≥ 80 cm for women and ≥ 85 cm for men) (34). The *P*-values for the interaction were also calculated by testing the multiplicative term of HGS and BMI or HGS and waist circumference. The sensitivity analyses were performed by defining the NAFLD using the HSI and FLI (21, 22). We then repeated the primary analyses by adjusting Model 2 in men and women. All the analyses were performed using the Statistical Analysis System (version 9.3 for Windows; SAS Institute, Inc., Cary, NC, USA). All the *P*-values were two-tailed, and the differences with *P*-values <0.05 were considered statistically significant.

## Results

### Participant Characteristics

The baseline characteristics of the participants according to sex are presented in Table 1. A total of 14,154 participants were enrolled. During the study period (mean follow-up, 3.20 years; range, 0.50–5 years), 2,452 participants (17.32%) developed NAFLD (incidence = 72.29 per 1,000 person-years). The proportion of men was 41.8%. The mean (SD) ages were 39.6 (11.5), 40.9 (12.5), and 38.7 (10.7) for all the participants, men, and women, respectively.

**Table 1:** The characteristics of participants by sex at baseline^a^.

**Characteristics**	**All**	**Men**	**Women**
	**(*n* = 14,154)**	**(*n* = 5,931)**	**(*n* = 8,223)**
No. of NAFLD^*^ in follow-up	2,452	1,534	918
Sex (male %)	41.8	-	-
Age (years)	39.6 (11.5)^b^	40.9 (12.5)	38.7 (10.7)
BMI	22.9 (3.0)	23.9 (2.9)	22.1 (2.8)
Waist circumference (cm)	78.4 (9.1)	84.2 (7.7)	74.3 (7.7)
Depressive symptoms score^c^	36.8 (7.8)	36.4 (7.8)	37.1 (7.7)
Physical activity (METs × hours/week)	19.6 (32.7)	23.1 (35.5)	17.1 (30.4)
Energy intake (kcal/d)	1,984.8 (837.6)	2,057.4 (885.2)	1,932.7 (797.7)
Education (≥College graduate, %)	69.3	69.2	69.5
Household income (≥10,000 Yuan, %)^d^	31.7	31.0	32.2
Dietary pattern scores (Multiplied by 10)			
Sweet pattern	0 (10)	−0.3 (10.6)	0.2 (9.5)
Vegetable pattern	0 (10)	0.8 (10.7)	−0.6 (9.4)
Animal foods pattern	0 (10)	1.3 (10.9)	−0.9 (9.2)
Smoking status (%)			
Smoker	14.4	33.4	0.9
Ex-smoker	3.7	8.1	0.5
Non-smoker	81.9	58.5	98.6
Drinker (%)			
Everyday	3.1	6.6	0.7
Sometime	53.1	72.0	39.7
Ex-drinker	9.0	9.5	8.6
Non-drinker	34.8	11.9	51.0
Employment status (%)			
Managers	46.6	46.7	46.4
Professionals	15.7	19.7	12.9
Other	37.7	33.6	40.7
Hypertension (%)	13.3	20.8	8.0
Hyperlipidemia (%)	32.5	36.2	29.8
Diabetes (%)	1.7	2.7	1.0

* *Non-alcoholic fatty liver disease (NAFLD) was diagnosed by ultrasonography and alcohol intake*.

a *NAFLD, non-alcoholic fatty liver disease; BMI, body mass index; MET, metabolic equivalent*.

b *Mean (SD) (all such values)*.

c *Assessed using Zung Self-rating Depression Scale*.

d *1 Yuan = 0.1555 dollar (2021-09-16 08:47)*.

### HGS and Incidence of NAFLD

As shown in Table 2, the baseline HGS [HGS/weight (kg/kg)] was negatively associated with the incidence of NAFLD in men (*P* < 0.0001) and women (*P* < 0.0001) before and after adjusting for the confounding factors. The multivariate-adjusted HRs (*CI*s) for NAFLD incidence across the quartiles of HGS were 1 (reference), 0.90 (0.79, 1.02), 0.69 (0.60, 0.79), and 0.44 (0.37, 0.52) for men and 1 (reference), 0.82 (0.69, 0.96), 0.54 (0.45, 0.66), and 0.41 (0.33, 0.52) for women. The optimal cut-off values of HGS (HGS/weight [kg/kg]) were 0.61 and 0.43 for men and women, respectively. The AUC (95% *CI*) values of HGS were 0.65 (0.63, 0.66) and 0.67 (0.66, 0.69) for men and women, respectively.

**Table 2:** Association between hand grip strength (HGS) (HGS/weight, kg/kg) and NAFLD^*^ by sex^a^.

	**Categories of hand grip strength (*****n*** **=** **14,154)**				***P* for trend^b^**
**Men**	**Level 1**	**Level 2**	**Level 3**	**Level 4**	
HGS (kg/kg)	0.17, 0.53	0.53, 0.60	0.60, 0.66	0.66, 1.23	
No. of participants	1,483	1,483	1,482	1,483	
No. of participants with NAFLD	560	449	331	194	
Crude model	Reference	0.81 (0.71, 0.92)^c^	0.58 (0.51, 0.67)	0.34 (0.28, 0.39)	<0.0001
Adjusted model 1^d^	Reference	0.90 (0.79, 102)	0.69 (0.60, 0.79)	0.44 (0.37, 0.52)	<0.0001
Adjusted model 2^e^	Reference	0.90 (0.79, 1.02)	0.69 (0.60, 0.79)	0.44 (0.37, 0.52)	<0.0001
Women	Level 1	Level 2	Level 3	Level 4	
HGS (kg/kg)	0.18, 0.39	0.39, 0.44	0.44, 0.50	0.50, 1.63	
No. of participants	2,057	2,055	2,055	2,056	
No. of participants with NAFLD	414	253	153	98	
Crude model	Reference	0.60 (0.52, 0.70)	0.36 (0.30, 0.43)	0.23 (0.19, 0.29)	<0.0001
Adjusted model 1^d^	Reference	0.80 (0.68, 0.94)	0.53 (0.44, 0.65)	0.40 (0.31, 0.50)	<0.0001
Adjusted model 2^e^	Reference	0.82 (0.69, 0.96)	0.54 (0.45, 0.66)	0.41 (0.33, 0.52)	<0.0001

* *NAFLD was diagnosed by ultrasonography and alcohol intake*.

a *HGS, hand grip strength; NAFLD, non-alcoholic fatty liver disease; BMI, body mass index*.

b *Multiple Cox regression analysis*.

c *Hazard ratios (95% CI) (all such values)*.

d * Adjusted for age and BMI*.

e *Adjusted for age, BMI, smoking status, drinking status, educational level, employment status, household income, physical activity, energy intake, type 2 diabetes, hypertension, hyperlipidemia, depressive symptoms, and intake of sweet foods pattern, vegetable pattern, and animal foods pattern*.

### BMI–HGS and Waist–HGS Interactions as Related to NAFLD Incidence

**Figure 2:**
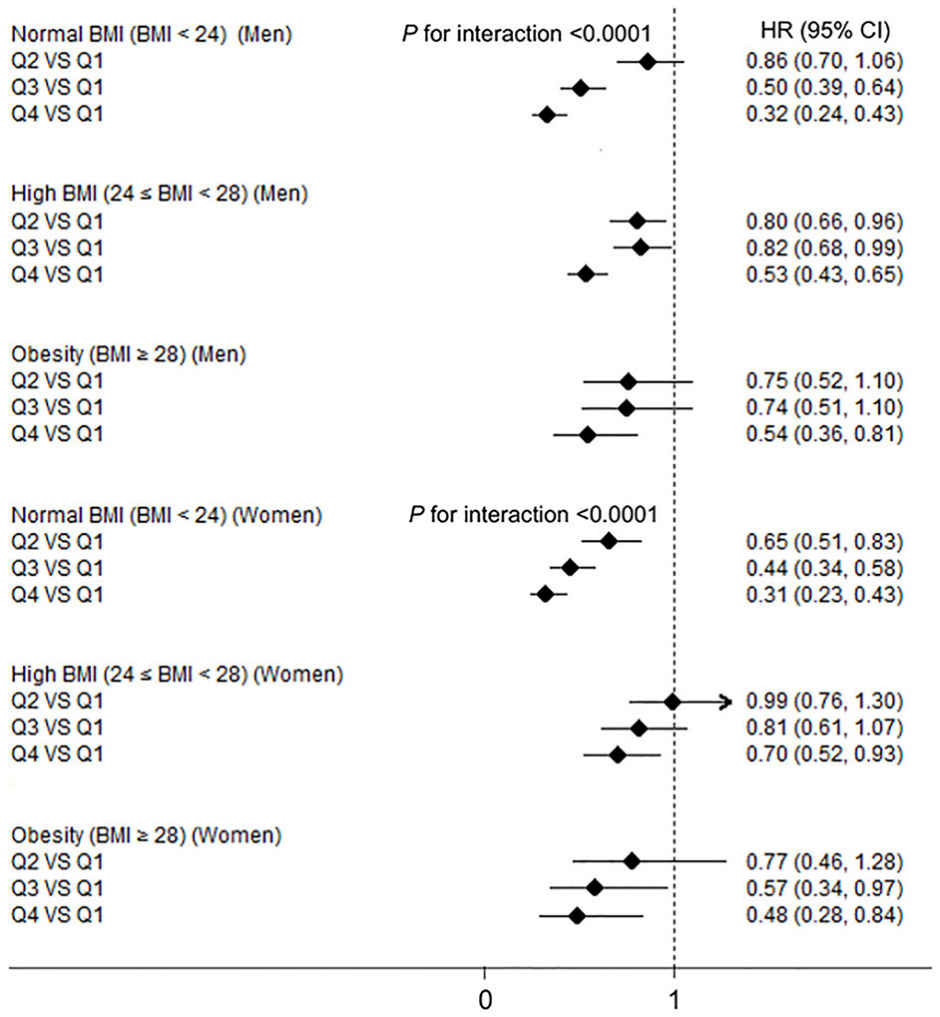
The associations between hand grip strength (HGS) and NAFLD according to body mass index (BMI) in men and women. NAFLD, non-alcoholic fatty liver disease; HGS, hand grip strength; BMI, body mass index; HR, hazard ratio; Q, quartile. Adjusted for age, smoking status, drinking status, educational level, employment status, household income, physical activity, energy intake, type 2 diabetes, hypertension, hyperlipidemia, depressive symptoms, and intake of sweet foods pattern, vegetable pattern, and animal foods pattern.

**Figure 3:**
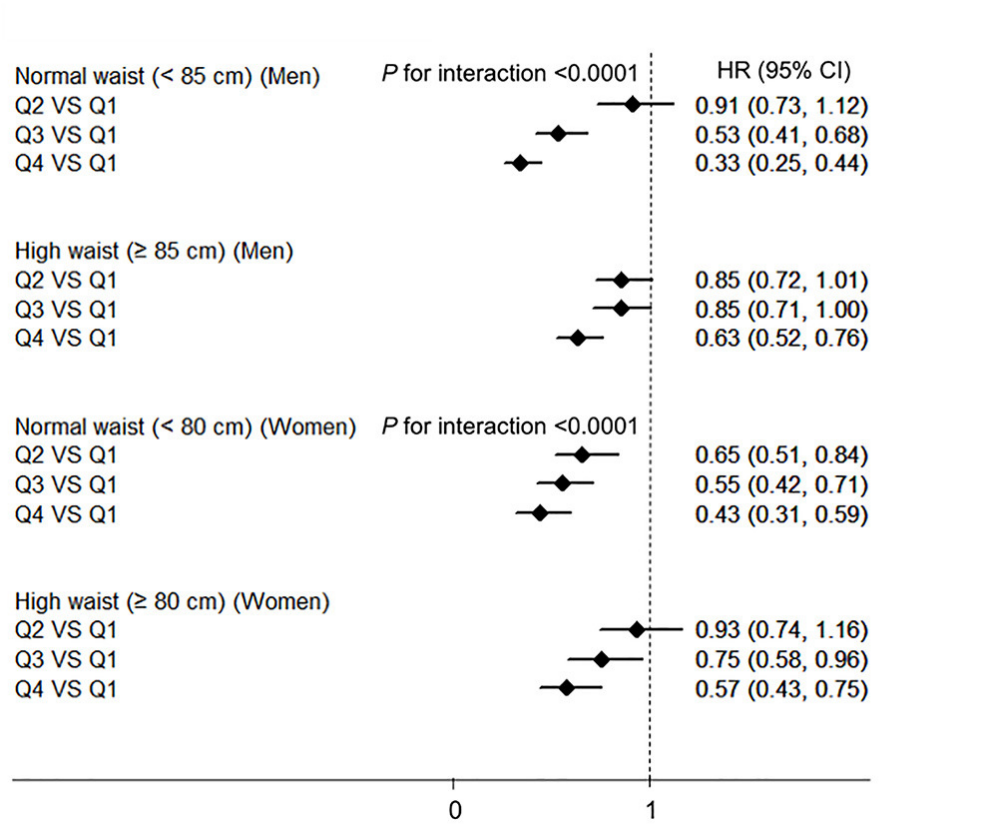
The associations between HGS and NAFLD according to the waist circumference in men and women. NAFLD, non-alcoholic fatty liver disease; HGS, hand grip strength; BMI, body mass index; HR, hazard ratio; Q, quartile. Adjusted for age, BMI, smoking status, drinking status, educational level, employment status, household income, physical activity, energy intake, type 2 diabetes, hypertension, hyperlipidemia, depressive symptoms, and intake of sweet foods pattern, vegetable pattern, and animal foods pattern.

The interaction terms of BMI–HGS and waist–HGS were significant in both men and women after adjusting for the confounding factors (all *P* < 0.0001). The associations among the different subgroups according to BMI and waist circumference are presented in Figures 2, 3, respectively. Compared with the participants in the lowest HGS quartiles, the lowest HRs (*CI*s) were observed among the participants who were in the normal BMI group (<24 kg/m^2^) in both men (HR: 0.32, 95% *CI*: 0.24, 0.43) and women (HR: 0.31; 95% *CI*: 0.23, 0.43), respectively. Similarly, in the subgroup analyses according to waist circumference, the associations between HGS and NAFLD were stronger in the participants who were in the normal waist group than those who were in the high waist group. Compared with the participants in the lowest HGS quartiles, the HRs (*CI*s) of NAFLD in the highest quartiles were 0.33 (0.25, 0.44) and 0.43 (0.31, 0.59) in men and women who had normal waist circumferences, respectively.

### Sensitivity Analysis

**Figure 4:**
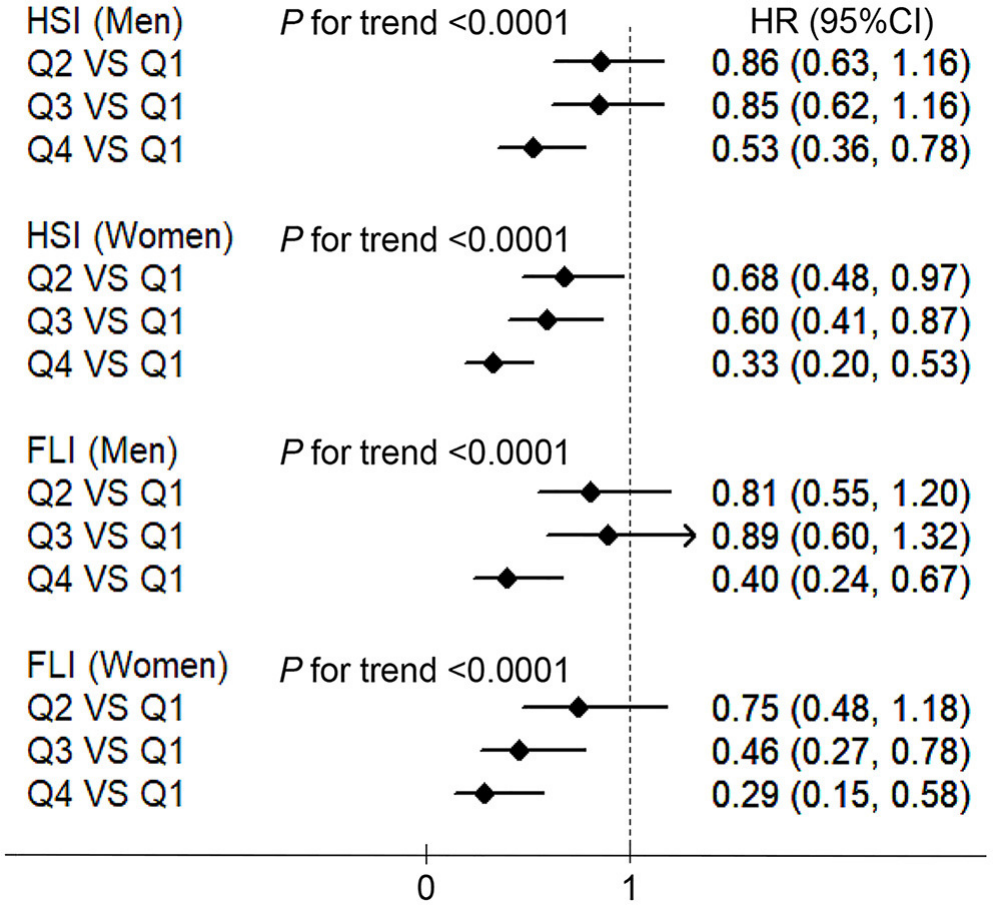
The associations between HGS and NAFLD which defined using the HSI and fatty liver index (FLI) in men and women. HSI, hepatic steatosis index; FLI, fatty liver index; NAFLD, non-alcoholic fatty liver disease; BMI, body mass index; HGS, hand grip strength; HR, hazard ratio; Q, quartile. Adjusted for age, BMI, smoking status, drinking status, educational level, employment status, household income, physical activity, energy intake, type 2 diabetes, hypertension, hyperlipidemia, depressive symptoms, and intake of sweet foods pattern, vegetable pattern, and animal foods pattern.

Figure 4 shows the association between the HGS and NAFLD incidence defined using the HSI and FLI in men and women. These associations were similar to those derived from the main analyses in which NAFLD was identified using liver ultrasonography and history of drinking. HGS was negatively associated with the incidence of NAFLD in both men and women (all *P* values <0.0001). Compared with the participants in the lowest quartiles of HGS, the HRs (*CI*s) of NAFLD which defined using HSI in the highest quartiles were 0.53 (0.36, 0.78) and 0.33 (0.20, 0.53) in men and women, respectively. Compared with the participants in the lowest quartiles of HGS, the HRs (*CI*s) of NAFLD which defined using FLI in the highest quartiles were 0.40 (0.24, 0.67) and 0.29 (0.15, 0.58) in men and women, respectively.

## Discussion

Our large population-based cohort study in which we prospectively determined the association between HGS and NAFLD in the Chinese adults suggested that a higher HGS was associated with a lower risk of NAFLD. These associations were independent of the socio-demographic, behavioral, psychological, dietary, and health status factors. Furthermore, there were significant interaction effects between HGS and both BMI and waist size on the incidence of NAFLD. The results of our sensitivity analysis, which defined NAFLD using the HSI and FLI, were similar to those of the main analysis.

The muscles have been shown to play an important role in the development of NAFLD (35). The previous studies found that sarcopenia, which is a progressive and generalized skeletal muscle disorder that involves the accelerated loss of muscle mass and function (36), was shown to be associated with NAFLD (37, 38). Compared with measuring muscle mass, measuring muscle strength (an indicator of muscle function) is easy to do in both the clinical and community settings (39). Thus, muscle strength could be a valuable predictor of NAFLD. In recent years, several cross-sectional studies have determined the association between HGS and the prevalence of NAFLD (13–17). For example, a cross-sectional study of 20,957 Chinese adults reported that increased HGS was independently associated with a lower prevalence of NAFLD (17). Compared with the participants who had the lowest HGS, the *OR* (95% *CI*) for the highest HGS was 0.67 (0.57, 0.79) (17). Another cross-sectional study also suggested that the low muscle strength was positively associated with NAFLD (*OR*, 1.47; 95% *CI*, 1.21, 1.80) in 5,132 Chinese adults (13). The Korea National Health and Nutrition Examination Survey also found that the lower BMI-adjusted HGS was associated with NAFLD in the Korean adults (*n* = 8,001) (16) as well as in elderly participants (*n* = 4,764) (14). Moreover, another study revealed a linear decrease in the NAFLD index that was commensurate with the incremental HGS level changes among the 538 elderly Korean participants (15). No cohort study has investigated the associations between HGS and NAFLD prospectively; considering the cross-sectional designs of previous studies, it was not possible to draw conclusions with respect to causality.

Consistent with previous cross-sectional studies, the present cohort study suggested that HGS was negatively associated with the incidence of NAFLD after adjusting for the confounding factors. The mechanisms that have been proposed to explain this association are chiefly related to insulin resistance and inflammation. First, the skeletal muscle is a major site of insulin-stimulated whole-body glucose disposal, and muscle metabolism can influence whole-body glucose homeostasis and insulin sensitivity (40). Thus, low muscle mass may lead to insulin resistance and explain the propagation of NAFLD (35). In addition, the muscle secretes irisin, which is a hormone that reduces obesity and insulin resistance (12), and is shown to be inversely associated with hepatic steatosis (41). Second, sarcopenia is associated with inflammatory indicators, such as the C-reactive protein level (38) and the neutrophil-to-lymphocyte ratio (42). Inflammation is also well-documented as a central component of NAFLD pathogenesis (43). Moreover, a previous study suggested that interleukin-6, which is a myokine secreted by muscle, was shown to have a protective effect on the development of NAFLD in an inflammation-prone animal model (44).

The results of the subgroup analyses suggested that the strongest associations between HGS and NAFLD were found in the participants with normal BMI and waist circumference. Two reasons were possible to explain it. First, BMI (45) and waist circumference (46) are positively associated with NAFLD. Thus, the associations between the HGS and NAFLD could be covered by BMI and waist circumference in the participants with high BMI and waist circumference. Second, the small sample sizes of high BMI and waist circumference subgroups could result in wide *CI*s of the HRs for NAFLD. We further performed the sensitivity analyses by defining NAFLD using the HSI and FLI. The associations revealed using this method were similar to those found when NAFLD was detected using liver ultrasonography and alcohol consumption history, indicating the robustness of the results.

The main strengths of our study were the large sample size and prospective cohort design. The former strength allowed for sufficient statistical power to detect the associations between HGS and NAFLD, while the latter strength helped ensure that the reverse causation would be minimized as much as possible. Moreover, the previous studies did not include dietary confounding factors in the adjustment models, even though the factors are strongly associated with muscle strength (47) and NAFLD (48). In the present study, we adjusted for sociodemographic, behavioral, psychological, dietary, and health status factors as much as possible to ascertain the independence from the association between HGS and NAFLD.

Some limitations are notable in our study. First, owing to its observational study design, the mechanism underlying the associations could not be determined. Second, even though we adjusted for the potential confounding factors, we could not rule out the possibility that other unmeasured factors might contribute to the associations observed. Third, we used hepatic ultrasonography scanning instead of the liver biopsies to detect fatty liver given that liver biopsy was not available during the health examinations of the target population. Even though a previous study found that ultrasonography had a sensitivity of 89% and a specificity of 93% for NAFLD and was widely used in population-based studies because of its non-invasiveness and accessibility (49), ultrasound has limited sensitivity and does not reliably detect steatosis when the amount of fat was low or in individuals with an elevated BMI. Thus, the patients from the NAFLD group (future NAFLD patients) may have NAFLD at the baseline but are diagnosed without NAFLD when using liver ultrasound. Future studies which use more accurate methods, such as liver biopsy and controlled attenuation parameter, are needed to confirm the observed associations in the present study. Fourth, we excluded participants with choric hepatitis B or C, autoimmune liver disease, and those who have previous liver surgery. Nevertheless, the participants with other causes of NAFLD (such as a drug), celiac disease, or thyroid disease were not excluded. Therefore, the observed associations may be affected. Finally, the mechanisms that underline the associations between HGS and NAFLD may be explained by the metabolic factors (e.g., insulin resistance). Otherwise, various factors play important roles in the development of NAFLD. For example, lean NAFLD was developed without obesity (50), and there is also a substantial proportion of patients with normal BMI NAFLD without insulin resistance. Moreover, the susceptible polygenic host background also contributes to the development of NAFLD (51). Therefore, despite metabolic factors, further studies should also focus on the effect of aforenoted factors on the development of NAFLD.

## Conclusion

Despite the aforementioned limitations, ours is the first cohort study to demonstrate that HGS is inversely associated with the incidence of NAFLD. The data suggested that a high HGS predicts a lower risk of NAFLD; hence, measuring HGS may serve as a possible strategy for detecting NAFLD at an early stage.

## Data Availability Statement

The raw data supporting the conclusions of this article will be made available by the authors, without undue reservation.

## Ethics Statement

The studies involving human participants were reviewed and approved the protocol of this study was approved by the Institutional Review Board of the Tianjin Medical University; the subjects provided written informed consent to participate in the study. The study protocol conformed to the Ethical guidelines of the 1975 Declaration of Helsinki. The patients/participants provided their written informed consent to participate in this study.

## Author Contributions

YX and KN contributed to the study conception and design. YX, LC, YL, XuW, SZ, GM, QZ, LL, HW, YG, YW, TZ, XiW, SS, MZ, QJ, and KS contributed to data collection, assembly, analysis, and interpretation of the data. YX, YZ, and KN contributed to the revising of the manuscript. YX, LC, and YZ contributed to the manuscript drafting and approval of the final version of the manuscript. All authors contributed to the article and approved the submitted version.

## Funding

This study was supported by grants from the National Natural Science Foundation of China (Nos. 81903302, 91746205, and 81673166) and 345 Talent Project of Shengjing Hospital of China Medical University (No. M0294).

## Conflict of Interest

The authors declare that the research was conducted in the absence of any commercial or financial relationships that could be construed as a potential conflict of interest.

## Publisher's Note

All claims expressed in this article are solely those of the authors and do not necessarily represent those of their affiliated organizations, or those of the publisher, the editors and the reviewers. Any product that may be evaluated in this article, or claim that may be made by its manufacturer, is not guaranteed or endorsed by the publisher.

## Abbreviations

AFLD: alcoholic fatty liver disease
AUC: area under the curve
BMI: body mass index
CI: confidence interval
FFQ: food frequency questionnaire
FLI: fatty liver index
HGS: hand grip strength
HIS: hepatic steatosis index
HRs: hazard ratios
MET: metabolic equivalent
NAFLD: non-alcoholic fatty liver disease
OR: odds ratio
Q: quartile
ROC: receiver operating characteristics
SDS: self-rating depression scale.
